# Malaria surveys using rapid diagnostic tests and validation of results using post hoc quantification of *Plasmodium falciparum* histidine-rich protein 2

**DOI:** 10.1186/s12936-017-2101-8

**Published:** 2017-11-07

**Authors:** Mateusz Plucinski, Rafael Dimbu, Baltazar Candrinho, James Colborn, Aida Badiane, Daouda Ndiaye, Kimberly Mace, Michelle Chang, Jean F. Lemoine, Eric S. Halsey, John W. Barnwell, Venkatachalam Udhayakumar, Michael Aidoo, Eric Rogier

**Affiliations:** 1 Malaria Branch, Division of Parasitic Diseases and Malaria, Centers for Disease Control and Prevention, Atlanta, GA USA; 2 U.S. President’s Malaria Initiative, Centers for Disease Control and Prevention, Atlanta, GA USA; 3 National Malaria Control Programme, Luanda, Angola; 4 National Malaria Control Programme, Maputo, Mozambique; 5 Clinton Health Access Initiative, Boston, MA USA; 6 Department of Pharmacy and Odontology, Cheikh Anta Diop University, Dakar, Senegal; 7 Programme National de Contrôle de la Malaria, Ministère de la Santé Publique et de la Population (MSPP), Port-au-Prince, Haiti

**Keywords:** Malaria, Rapid diagnostic test, Limit of detection, Bead assay, Histidine-rich protein 2

## Abstract

**Background:**

Rapid diagnostic test (RDT) positivity is supplanting microscopy as the standard measure of malaria burden at the population level. However, there is currently no standard for externally validating RDT results from field surveys.

**Methods:**

Individuals’ blood concentration of the *Plasmodium falciparum* histidine rich protein 2 (HRP2) protein were compared to results of HRP2-detecting RDTs in participants from field surveys in Angola, Mozambique, Haiti, and Senegal. A logistic regression model was used to estimate the HRP2 concentrations corresponding to the 50 and 90% level of detection (LOD) specific for each survey.

**Results:**

There was a sigmoidal dose–response relationship between HRP2 concentration and RDT positivity for all surveys. Variation was noted in estimates for field RDT sensitivity, with the 50% LOD ranging between 0.076 and 6.1 ng/mL and the 90% LOD ranging between 1.1 and 53 ng/mL. Surveys conducted in two different provinces of Angola using the same brand of RDT and same study methodology showed a threefold difference in LOD.

**Conclusions:**

Measures of malaria prevalence estimated using population RDT positivity should be interpreted in the context of potentially large variation in RDT LODs between, and even within, surveys. Surveys based on RDT positivity would benefit from external validation of field RDT results by comparing RDT positivity and antigen concentration.

**Electronic supplementary material:**

The online version of this article (10.1186/s12936-017-2101-8) contains supplementary material, which is available to authorized users.

## Background

Malaria rapid diagnostic tests (RDTs) have now been in use for nearly 20 years, and were intentionally designed for portability, ease of use, and reliability in resource-limited settings. Following the recommendations of the World Health Organization (WHO) for universal confirmation of all malaria infections before treatment, demand for malaria RDTs has grown substantially with an estimated 314 million tests procured in 2015, the vast majority of these tests designed to detect the *Plasmodium falciparum* histidine-rich protein 2 (HRP2) [1]. This practical tool has allowed malaria control programmes to more accurately characterize malaria burden and adopt policies to move beyond presumptive treatment to allow confirmation of infection prior to initiation of treatment. Malaria confirmation by RDT has led to more responsible use of anti-malarial drugs [2, 3], which may help reduce drug pressure and thus the potential for *P. falciparum* anti-malarial resistance. Additionally, RDTs have eclipsed microscopy in many areas; the roll back malaria monitoring and evaluation reference group recently released guidelines stating that national household surveys such as the demographic and health survey or malaria indicator survey could use RDTs alone to measure malaria prevalence [4].

HRP2 is only produced by one of the human malarias, *P. falciparum* [5], and assaying for this protein provides a species-specific malaria test. HRP2 concentration in malaria infections can vary over orders of magnitude even in infections with the same parasite density due to differences in parasite production of HRP2 and host clearance of HRP2, and can persist for weeks following parasite clearance [6]. Annual product testing of RDTs performed by the WHO in conjunction with the foundation for innovative new diagnostics (FIND) and centers for disease control and prevention (CDC) has allowed for standardized analysis and reporting of RDT quality and performance from a variety of different manufacturers [7, 8]. Currently, HRP2-based RDTs are assessed against a panel of well-characterized culture-derived *P. falciparum* strains and wild isolates collected from several malaria endemic countries and diluted to 200 and 2000 parasites/µL for which HRP2 protein concentrations are known. These data quantifying HRP2 content of the panel samples are used primarily to standardize year to year sample set composition leaving parasite density as the primary sample characteristic. Product performance is estimated for samples at 200 and 2000 parasites/µL with performance at 200 parasites/µL used as the ultimate determinant of product quality, with the objective of ensuring good performance in the clinical setting. Product specificity determination involves testing products on multiple known HRP2 negative blood samples, including samples containing molecules or antibodies that could cross-react with the test reagents on the RDT filter strip, potentially providing false-positive results [8].

Although RDTs were standardized for use in case management and their operational sensitivity is expected to be around 100 parasites/µL, use of RDTs for various malaria surveys including burden estimates require clear definition of their detection limit and other operational characteristics in the field settings. Moreover, the range of HPR2 concentrations in survey settings are likely to differ from those in the clinical setting, and the performance of RDT results in this setting has not been evaluated. Unlike blinded external quality control of microscopy slides, there is currently no accepted method for external validation of RDT results for various malaria surveys. The recent development of a bead-based HRP2 assay that can detect HRP2 concentrations in the single picogram range [9] opens up the possibility for a reference assay which would detect HRP2 concentrations orders of magnitude below the capacity of a conventional RDT. Using this method, dried blood samples from individuals previously receiving a RDT during surveys were tested in the laboratory to validate RDT results obtained in the field. Samples representing a wide range of human and *P. falciparum* populations, from Angola, Mozambique, Senegal, and Haiti were assayed for HRP2 concentration. Samples came from surveys conducted in areas of low and high *P. falciparum* transmission, and in community and health facility settings. This strategy introduces an applied method for assessing the true performance of a RDT in situ and thus providing an external validation for RDT results from malaria surveys.

## Methods

### Sample collection

Previously-collected, anonymized samples from six surveys from Angola [10], Mozambique [7], Haiti, and Senegal [11] were tested on the novel HRP2 assay platform (Table 1). In each survey, participants were administered an RDT and had blood collected on filter paper. The RDTs used included SD Bioline *Pf/Pv* (Standard Diagnostics, Yongin, Republic of Korea) in the Angola surveys, SD Bioline Malaria Ag *Pf* (Standard Diagnostics, Yongin, Republic of Korea) in the Mozambique surveys, First Response Ag *Pf* (HRP2) Card Test (Premier Medical Corporation, Denver, USA) in Haiti, and *CareStart™* Malaria HRP2/pLDH(*Pf*/PAN) Combo (Access Bio, Somerset, USA) in Senegal. Dried blood spots were collected on Whatman 903 protein saver cards (GE Healthcare Life Sciences, Pittsburgh, USA) in the Angola and Haiti surveys, on TropBio filter paper (Cellabs, Brookvale, Australia) in Mozambique, and on Whatman FTA cards (GE Healthcare Life Sciences, Pittsburgh, USA) in Senegal.

**Table 1:** Study design and study population of surveys analysed to assess field HRP2-based RDT performance

	Survey					
	Angola Huambo	Angola Uíge	Mozambique 2013	Mozambique 2014	Haiti	Senegal
Persons sampled	607	647	1064	1015	4350	501
Period	2016, rainy season	2016, rainy season	2013, dry season	2014, dry season	2014–2015	2015
Median age (range)	14 (< 1–90)	16 (< 1–90)	16 (< 1–89)	11 (< 1–86)	25 (< 1–99)	23 (3–77)
Population	Febrile and afebrile patients attending health facilities	Febrile and afebrile patients attending health facilities	Community sample	Community sample	Community sample	Febrile patients attending 2 health facilities
% RDT-positive^a^ (%)	11	46	57	68	1	68
Type of RDT	SD bioline Pf/Pv	SD bioline Pf/Pv	SD bioline Pf	SD bioline Pf	First response Pf	CareStart Pf/Pan

*RDT* rapid diagnostic test, *HRP2* histidine-rich protein 2

^a^Excluding Pv-positive only for the Angolan surveys

The six surveys varied in geographical scope, patient population, and malaria endemicity (Table 1). The Mozambique and Haiti surveys were household surveys where all consenting household members of all ages, regardless of symptoms, were sampled. In contrast, the Angola and Senegal surveys were performed at health facilities. In Angola, randomly selected outpatients of all ages were invited to participate regardless of symptoms, whereas in Senegal febrile patients of all ages were sampled.

Testing of samples was covered by the original study protocols for Angola, Mozambique, and Haiti, which were reviewed and approved by the Angolan Ministry of Health (MOH), the Mozambique National Bioethics Review Board, and the Haitian MOH, respectively. Additional testing of stored anonymized samples from the Senegal survey was reviewed and approved as non-research by the CDC center for global health human subjects office.

### Detection and quantification of HRP2 by the bead assay

The HRP2 bead assay was performed as described previously [9]. Monoclonal IgM (MPFM-55A, Abcam; ab9206) antibodies were covalently bound to polystyrene BioPlex^®^ COOH beads (BioRad; 1715060XX) by the commonly-used EDC/Sulfo-NHS intermediate reaction at a concentration of 20 μg antibody/12.5 × 10^7^ beads. Reactive esters were formed on the carboxylated beads in the presence of EDAC [1-Ethyl-3-(3ʹ-dimethylaminopropyl)carbodiimide](EMD Millipore; 341006) and Sulfo-NHS [*N*-hydroxysulfosuccinimide] (ThermoScientific; 24510) under light agitation for 20 min. Carboxyl to antibody amine cross-linking occurred in activation buffer (LuminexCorp; 11-25171) under light agitation for 2 h. Nonspecific protein binding was blocked by incubation with BSA (PBS pH7.2, 0.05% Tween20 [PBS-T] + 1% BSA) for 30 min; beads were then resuspended in blocking buffer with 0.02% NaN_3_. Dried blood spot samples were eluted in PBS-T containing 0.02% NaN_3_ to a final concentration of 1:20 × whole blood. Reagent diluent consisted of PBS-T plus 0.5% BSA, 0.02% NaN_3_. Filter bottom plates (Millipore; MABVN1250) were pre-wetted with PBS-T. Approximately 1500 coupled beads were incubated with sample for 1.5 h under gentle shaking. Wells were washed with PBS-T (3 × between all incubations), and incubated for 45 min with 50uL biotinylated detection antibody (1:250 ×, mouse IgG anti-HRP2, MPFG-55P, Abcam; ab9203) (antibody previously biotinylated by ThermoScientific EZ-Link Micro Sulfo-NHS-Biotinylation Kit according to manufacturer’s protocol; 21925). Beads in wells were subsequently incubated with 50 μL streptavidin–phycoerythrin (1:100 ×, invitrogen; S866) for 30 min. Beads in wells had a final wash incubation with 50 μL reagent diluent for 30 min, were resuspended in 100 μL PBS, and were read on a Bio-Plex 200 machine (BioRad; 171000201) by generating the median fluorescence signal for 50 beads and then the mean fluorescence intensity (MFI) of the medians among replicates. The final measure, denoted as MFI-bg, was reported by subtracting MFI values from blank control wells (beads exposed to only sample diluent during the sample incubation step).

### Statistical analysis

For each survey, a non-parametric LOESS curve [12] was generated to characterize the relationship between the likelihood of a positive RDT in the field and the log HRP2 concentration measured in the laboratory. A logistic regression model was fit to the dose–response data, and was used to estimate the HRP2 concentrations with 95% confidence intervals at which 50, 75, 90, and 95% of the RDTs would be expected to turn positive (level of detection [LOD]). A multivariate regression model was fit to explore the relationship between participant age and sex and testing RDT positive, after adjustment for HRP2 concentration.

## Results

**Fig. 1:**
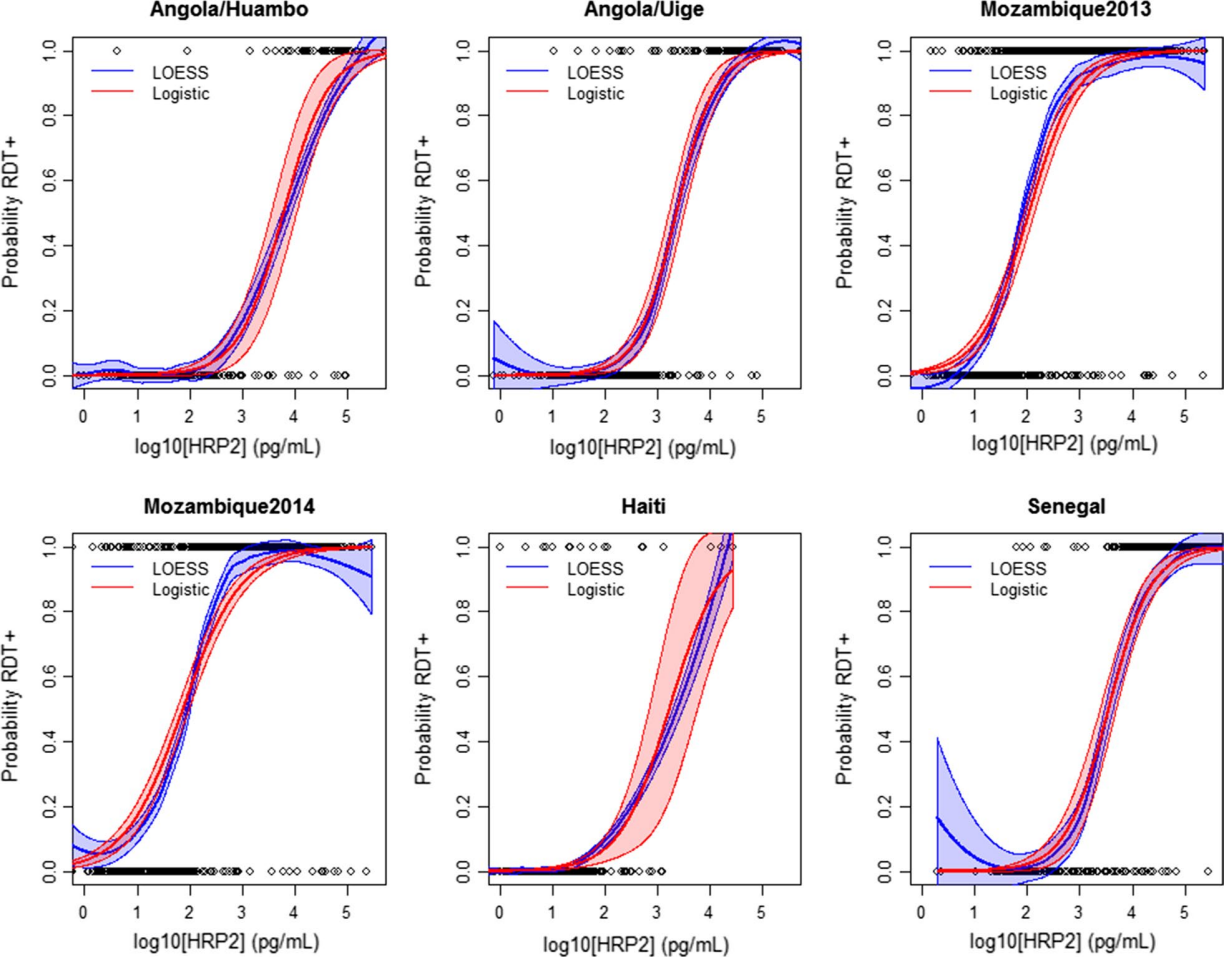
Relationship between the probability of testing RDT positive and log HRP2 concentration, assessed for six different surveys from sub-Saharan Africa and Haiti. Shaded areas represent the 95% confidence intervals of the LOESS and logistic models

A total of 8184 individuals from six surveys were given a HRP2-based RDT and had their blood sample quantified for HRP2 concentration. The relationship between RDT positivity and log_10_ HRP2 concentration was sigmoidal in all six surveys, and a logistic dose–response model provided a good fit for all datasets (Fig. 1). The estimated slope parameter, controlling the shape of the dose–response curve, was not statistically different between the six surveys (Additional file 1), but there was considerable variability in the coefficient determining the position of the inflection point. This was reflected in differences in the estimated LODs, which varied significantly by survey (Table 2). The 50% LOD ranged from 0.076 ng/mL (95% CI 0.057–0.098) in the Mozambique 2014 survey to 6.1 ng/mL (3.6–11) in the Angola Huambo survey. A similar pattern was observed in the 95% LOD, which varied from 2.5 ng/mL (1.5–3.6) in the Mozambique 2013 survey to 109 ng/mL (37–225) in the Angola Huambo survey. The LOD was significantly higher in the Angola survey in Huambo Province compared to Uíge Province. For the samples from Mozambique, the 95% confidence intervals overlapped between the 2013 and 2014 surveys. The Angola Uíge, Haiti and Senegal surveys had largely overlapping confidence intervals for the different LODs.

**Table 2:** Estimated in situ level of detection of HRP2-based rapid diagnostic tests used in six field surveys, as estimated from logistic dose–response model

Sensitivity (%)	HRP2 concentration (ng/mL)					
	Angola Huambo	Angola Uíge	Mozambique 2013	Mozambique 2014	Haiti	Senegal
50	6.1 (3.6–11)	2.3 (1.6–3.2)	0.11 (0.087–0.14)	0.076 (0.057–0.098)	1.7 (0.7–5.8)	3.6 (2.4–5)
75	18 (9.4–32)	5.8 (3.9–8.3)	0.35 (0.26–0.46)	0.3 (0.22–0.41)	5.6 (1.5–18)	10 (7–14)
90	53 (22–101)	15 (9–22)	1.1 (0.75–1.6)	1.2 (0.81–1.8)	18 (2.7–62)	28 (18-41)
95	109 (37–225)	28 (15–43)	2.5 (1.5–3.6)	3.2 (1.9–4.9)	41 (3.6–145)	57 (33–88)

Numbers in parentheses represent 95% confidence intervals

Age and gender were significantly correlated with the likelihood of testing RDT positive, even after adjusting for HRP2 concentration. Compared to children < 5 years of age, persons > 15 years of age were less likely to test RDT positive (adjusted odds ratio 0.6, 95% CI 0.4–0.9) (Table 3). Women were also less likely to test RDT positive than men with the same HRP2 concentration (adjusted odds ratio 0.7, 95% CI 0.6–1.0).

**Table 3:** Factors influencing sensitivity of HRP2-based RDT performance as assessed using multivariate logistic regression across five surveys

	Odds ratio of testing positive by RDT	
	aOR	95% CI
Log HRP2 concentration (pg/mL)	8.6	(8–10)
Age		
< 5	Ref.	
5–14	1.2	(0.8–2)
≥ 15	0.6	(0.4–0.9)
Female	0.7	(0.6–1)

*RDT* rapid diagnostic test, *HRP2* histidine-rich protein 2

## Discussion

In all six surveys RDTs were consistently able to detect a wide range of antigenaemia concentrations. RDTs are designed and tested to be able to detect clinically relevant antigen concentrations, and the estimated LODs in all six surveys were consistent with the HRP2 concentrations in panels used by WHO/CDC product testing [8]. However, there was significant variation among surveys in the ability of the RDTs to detect HRP2 at lower concentrations. The differences were observed not just in surveys in different countries using different RDTs, but even between different areas in the same country as evidenced in the case of the Angola surveys where there was a threefold difference in the LOD estimates. Furthermore, data from the Mozambique community surveys provided very low LOD estimates at the 95% sensitivity level of 2.5 and 3.2 ng/mL for 2013 and 2014, respectively. These data indicate that HRP2-based RDTs could potentially be detecting much lower concentrations of HRP2 than had been anticipated based on expectations that had been generated from standardized WHO/CDC testing of high HRP2 concentrations. While the differences in LOD observed here do not impact the interpretation of RDT results in a clinical setting [13], where existing RDTs adequately detect antigen levels associated with acute malaria cases, differences in the lower level of detection of RDTs will have an impact when RDTs are used for measuring and tracking malaria burden at the population level. For example, 14% of the tested samples included in the analysis reported here had an HRP2 concentration between 1.11 and 53 ng/mL, the range of 90% LODs observed in the surveys, representing the range of antigen concentrations that could potentially have different RDT results depending on survey conditions. However, without an independent measure of parasitaemia for the samples analysed here, either through microscopy or PCR, it is not possible to estimate how this difference in LODs would influence how well RDT positivity reflects parasite prevalence in the population.

Contrary to the performance that is evaluated during routine product testing, which aims to measure an intrinsic characteristic of the RDT and systematically compare performance among dozens of manufactured tests, the field performance of an RDT is subject to a multitude of factors. These can be divided into three categories. First, the RDT performance is dependent on the quality of the production run for a particular lot, an intrinsic property of the test itself, which would be expected to vary between different manufacturers, different products from the same manufacturer, and different lots of the same product [14]. Second, the performance will be influenced by the field conditions preceding and during its administration: the storage of the RDT, the training and supervision of the operator, the setting of its use, and the visual acuity of the operator. Pre-test probability, the operator’s a priori expectation regarding the result of the test, is part of these factors, and could be a potential explanation for the finding that children and women had higher likelihood of testing RDT positive at the same HRP2 level as older children and adults, and men, respectively. Third, characteristics of the host and parasite population could affect the RDT performance. Heterogeneity in HRP2 size (and epitope number) has been widely hypothesized to play a role in reliability of RDT tests [15–17], but the variables of *Pfhrp2* transcription levels [18] and host antibodies [19] may also affect field test results. Additionally, direct comparison of the RDT LODs estimated here among populations in separate surveys is limited due to inherent differences in filter paper, collection and storage procedures. Although sources of error can also arise in laboratory assays, inter-assay variation for the bead assay was minimized by use of a single bead coupling and assay reagents for all studies and the same standard for all calculations of HRP2 concentrations.

Regardless of the underlying reason for differences in the estimated LOD for RDTs performed in the field, our results suggest that caution is needed when comparing RDT positivity rates across sites, periods, and parasite and host populations. Ultimately, population RDT positivity is a measure of the prevalence of malaria antigenaemia, and not a measure of parasite prevalence. Direct ways of measuring parasite prevalence include microscopy and PCR, but both of these techniques are themselves subject to factors influencing between-survey heterogeneity in sensitivity. As we show here, the level at which RDTs are able to detect malaria antigenaemia is not uniform, and numerous factors could lead to different test performance in different settings. As a result the use of population RDT positivity as an empirical measure for *P*. *falciparum* transmission intensity should interpreted in the appropriate context. The recent shift from measuring malaria prevalence through microscopy to RDTs has been motivated by the ease of use of RDTs and their perceived robustness in field settings. Whether it is for measuring impact by comparing changes in RDT prevalence, or stratifying malaria risk geographically by comparing RDT positivity in different regions, a crucial assumption is that RDT results can be compared across time and space. However, as suggested by the results presented here, additional thought should be given to a system for external validation of in situ RDT results for a sample population. For example, the interpretation of an RDT-positive result in the two surveys in Angola, which shared the same methodology, brand of RDTs, and sample collection procedures, was different between two provinces, with a threefold difference in estimated LODs between them. Inter-lot variation, product storage, operator performance, host genetics, or parasite genetics are some of the many potential factors that could account for the difference in the estimate of the LOD in the two provinces. Additionally, as the laboratory-based bead assay is orders of magnitude more sensitive than field RDT tests, detection of the HRP2 antigen in individuals’ blood samples would provide an additional benefit of estimating the overall RDT false positivity rate. Though blood dried on specified filter paper was used exclusively as the sample type in this study, other sample types would also be able to be accommodated, potentially even the filter strip in an RDT itself. Barring an error in the bead assay results, individuals found to not have any HRP2 by the bead assay would either have a complete absence, or such a low amount, of HRP2 that it would be very unlikely that they would test positive by an RDT. This quality assurance may prove especially useful in areas of low transmission, where few positives would be expected from a survey, and the false positives can substantially bias the final result.

## Conclusions

The ability to characterize the HRP2 concentration from dried blood spots in a high-throughput manner allows the possibility of providing extra context for binary RDT results. Future surveys where RDT positivity is used as a primary indicator of malaria burden would benefit from the collection of dried blood spots in at least a subsample of the population for measurement of HRP2 concentrations in the laboratory and retrospective validation. In this way, the field performance of the RDT, in the form of the dose–response curve and estimated LODs, could be reported together with the RDT indicators to aid in interpretation of survey results. In order to establish a standardized validation protocol there is need to develop common standards for multiplex bead assays, standardized protocols for blood spot collection, agreed criteria for interpretation of bead assay results, collaboration between international partners, and guidance from WHO.

## Additional file

**Additional file 1: Table S1.** Point estimates and 95% confidence intervals for logistic dose-response model fit to data on rapid diagnostic test positivity as a function of HRP2 concentration in six field surveys

## Abbreviations

CDC: the centers for disease control and prevention
HRP2: histidine-rich protein 2
LOD: limit of detection
RDT: rapid diagnostic test
WHO: World Health Organization
