# Supplementary material for: Malaria surveys using rapid diagnostic tests and validation of results using post hoc quantification of Plasmodium falciparum histidine-rich protein 2
Source: Malar J. 2017 Nov 7;16:451. doi: 10.1186/s12936-017-2101-8 (PMC5678810; doi:10.1186/s12936-017-2101-8)
Supplement: Supplementary file 1 — Additional file 1: Table S1. Point estimates and 95% confidence intervals for logistic dose-response model fit to data on rapid diagnostic test positivity as a function of HRP2 concentration in six field surveys [file 12936_2017_2101_MOESM1_ESM.pdf]

Table S1. Point estimates and 95% confidence intervals for logistic dose-response model fit to data on rapid diagnostic test positivity as a function of HRP2 concentration in six field surveys

Coefficient	Angola Huambo	Angola Uíge	Mozambique 2013	Mozambique 2014	Haiti	Senegal
β_0	-8.9 (-11.2--7.2)	-9.1 (-10.8--7.8)	-4.4 (-5.0--3.9)	-3.4 (-3.9--2.9)	-6.8 (-7.9--6.0)	-8.7 (-11--7.1)
β_1	2.4 (1.9-2.9)	2.7 (2.3-3.2)	2.2 (1.9-2.4)	1.8 (1.6-2.0)	2.1 (1.6-2.7)	2.4 (2-2.9)

$$\frac{P(RDT+)}{1-P(RDT+)} = \exp(\beta_0 + \beta_1 \log[\mathrm{HRP2}])$$
